# Characterization of Prophages in *Leuconostoc* Derived from Kimchi and Genomic Analysis of the Induced Prophage in *Leuconostoc lactis*

**DOI:** 10.4014/jmb.2110.10046

**Published:** 2021-12-23

**Authors:** Song-Hee Kim, Jong-Hyun Park

**Affiliations:** Department of Food Science and Biotechnology, College of BioNano Technology, Gachon University, Seongnam 13120, Republic of Korea

**Keywords:** Kimchi, *Leuconostoc*, prophage, immunity gene, prophage induction, *Leu. lactis*

## Abstract

*Leuconostoc* has been used as a principal starter in natural kimchi fermentation, but limited research has been conducted on its phages. In this study, prophage distribution and characterization in kimchi-derived *Leuconostoc* strains were investigated, and phage induction was performed. Except for one strain, 16 *Leuconostoc* strains had at least one prophage region with questionable and incomplete regions, which comprised 0.5–6.0% of the bacterial genome. Based on major capsid protein analysis, ten intact prophages and an induced incomplete prophage of *Leu. lactis* CBA3626 belonged to the *Siphoviridae* family and were similar to Lc-Nu-like, sha1-like, phiMH1-like, and TPA_asm groups. Bacterial immunology genes, such as superinfection exclusion proteins and methylase, were found on several prophages. One prophage of *Leu. lactis* CBA3626 was induced using mitomycin C and was confirmed as belonging to the *Siphoviridae* family. Homology of the induced prophage with 21 reported prophages was not high (< 4%), and 47% identity was confirmed only with TPA_asm from *Siphoviridae* sp. isolate ct3pk4. Therefore, it is suggested that *Leuconostoc* from kimchi had diverse prophages with less than 6% genome proportion and some immunological genes. Interestingly, the induced prophage was very different from the reported prophages of other *Leuconostoc* species.

## Introduction

Lactic acid bacteria (LAB) in the kimchi microbiota are well-known players in fermentation and contribute to the sensory attributes and preservation of kimchi. However, the roles of bacteriophages (phages) originating from LAB in the microecosystem and the virome during the fermentation process are still unknown. Recently, phages from kimchi metagenomic research have been estimated to exist in large numbers and coexist stably with the bacterial commensals [1]. The phage community may also have potential roles in fermentation of LAB-related foods such as cheese, sausage, sauerkraut, and cucumber [2, 3].

Kimchi is a traditional Korean fermented food made of vegetables and various other ingredients. Abundant bacteria exist in kimchi, and *Leuconostoc* is one of the predominant bacteria that usually appears in the early and middle stages of kimchi fermentation [4]. *Leuconostoc* produces substances that are responsible for the desirable flavor and taste of kimchi. In the kimchi industry, starters are used to achieve a uniform taste and fast fermentation. However, problems can be encountered, sometimes causing failures. Several factors may contribute to these problems, and the existence of phages might be a reason for these failures. Although several studies have been conducted on phages of LAB starters in the dairy industry, there has been little research on phages during plant-based natural fermentation, such as kimchi [5, 6]. The dominance of phages has been confirmed using metagenomic analysis, and some reports have shown that temperate phages are dominant in kimchi [7, 8]. These studies have suggested that the phage population increases during kimchi fermentation, consequently affecting the process [1, 9]. Recently, the phage population in watery kimchi has been found amounting to approximately 30% of bacterial counts on a log scale and fluctuating during fermentation [10, 11].

Viruses are the most abundant and ubiquitous entities on our planet; they are present with an estimated number of 10^31^ particles, the majority of which are phages. Phages infect specific bacterial species or strains and disrupt bacterial metabolism [12, 13]. Most phages have double-stranded DNA and belong to the order Caudovirales; they are mainly observed in families *Myoviridae*, *Siphoviridae*, and *Podoviridae*, and identified using morphological analysis [14]. The roles of phages in the environmental ecosystem have been speculated to be specific predation on microbial diversity and governance of population dynamics through lysogenic and lytic life cycles. Daily prokaryotic mortality of 20–50%, which may be a major source of the dissolved organic matter in nature, is estimated to originate from viral infections [15, 16]. In addition to the virulent phages released after cell lysis, some phage genes are incorporated into the bacterial genomes by 10–20% as prophages, which are major contributors to the differences between bacteria, even within a species [17]. Through phage transduction, hosts often obtain foreign genes for resistance to environmental stresses and coexistence with phages [18, 19]. Such events may affect bacterial ecology in terms of population changes in the microecosystem and contribute to the adaptation and evolution of microbial populations in natural environments [20].

Studies on phages during fermentation are required to determine whether they truly modulate kimchi fermentation or simply reflect the compositional changes in the bacterial community. Some studies on kimchi-derived LAB phages have been performed; however, no studies on prophages present in the genomes have been conducted yet. Therefore, our aim in this study was to identify the prophage composition in kimchi-derived *Leuconostoc* genomes and compare them with other phages. Identifying and characterizing the phages of *Leuconostoc*, a major kimchi starter, might provide a better understanding of LAB ecology in the kimchi environment.

## Materials and Methods

### *Leuconostoc* spp. Strains and Growth Conditions

Eight bacterial strains of *Leuconostoc* were examined in this study (as shown in Table 1, with asterisks). The strains were inoculated at 1% (v/v) into de Man, Rogosa, and Sharpe (MRS) media (Oxoid, England) and cultured at 30°C for 24 h. Stock cultures were stored in 20% glycerol at −80°C.

### Prophage Identification

The complete genome information of kimchi-derived *Leuconostoc* strains was downloaded from the Pathosystems Resource Integration Center (PATRIC) [21]. Based on the sequence data from PATRIC, prophage-integrated regions were analyzed using PHAge Search Tool Enhanced Release (PHASTER) [22]. PHASTER provides information on the completeness of the predicted phage-related regions according to the number of known genes/proteins contained in the bacterial prophage region: intact (>90%), questionable (90%–60%), and incomplete (<60%) regions. A prophage analysis tool, Prophage Hunter [23], was also used for further analysis of *Leu. lactis* CBA 3626.

### Phylogenetic Analysis

The major capsid protein (MCP) sequences of intact *Leuconostoc* prophages and similar phages were aligned using ClustalW [24]. Phylogenetic trees were constructed using the neighbor-joining method of the MEGA7 software program [25].

### Morphology and Phage-Encoded Resistance System Identification

Superinfection exclusion (Sie) proteins were manually annotated as described previously [26]. Briefly, between the integrase and repressor of the prophages, proteins having one or more N-terminal transmembrane domains were predicted using the TMHMM Server, v. 2.0 [27] and protein adjacent to the metalloprotease and the metalloproteases were identified as Sie proteins. Methylase (MTase) proteins were predicted using BLASTp searches [28].

### Prophage Induction and Validation

Overnight cultures of *Leu. lactis* CBA 3626, *Leu. citreum* CBA 3621, and *Leu. citreum* CBA 3627 were inoculated at 1% (v/v) on fresh MRS broth and incubated at 30°C until an OD_600_ reading of 0.2 was achieved. Then, mitomycin C (MitC) (Sigma-Aldrich, USA) was added to a final concentration of 0.2, 0.5, and 1 μg/ml [29]. MitC-treated culture and control (MitC non-treated) were grown and observed for 24 h, and the absorbance at OD_600_ was measured every 2 h. Subsequently, the culture broth was centrifuged at 8,000 ×*g* at 4°C for 10 min, and the supernatant was filtered through a 0.22 μm filter (Millipore, USA). The filtered supernatants were concentrated through centrifugation at 26,000 ×*g* for 1 h.

To confirm prophage induction, spotting assay and transmission electron microscopy (TEM) were performed. For the spotting assay, 100 μl of each *Leuconostoc* overnight culture was inoculated in 5 ml MRS soft agar (0.7%agar) and overlaid on MRS agar. Then, 10 μl of the concentrated supernatant was spotted on the lawn and incubated overnight at 30°C to observe the lysis zone [8].

To observe phage morphology using TEM, the concentrated supernatants were inoculated on a 200-mesh, carbon-coated copper grid (Ted Pella, USA) and stained with 2% uranyl acetate. The samples were observed using TEM (H-7600, Hitachi, Japan) at 80 kV [30].

To detect the induced phage using polymerase chain reaction (PCR), the primers for the MCPs of intact, incomplete, and questionable prophages of *Leu. lactis* CBA3626 were designed (as listed in Table S1). The primers for the MCP, endolysin, and tail proteins of the two fused, incomplete prophages of *Leu. lactis* CBA3626 are listed in Supplementary Table 2. The housekeeping gene glyceraldehyde 3-phosphate dehydrogenase was used as a control. Each concentrated supernatant was treated with DNase for 30 min at 37°C and inactivated at 75°C for 10 min to remove bacterial DNA. According to the manufacturer’s protocol, 5 μl of the supernatant was used as the PCR template, and AccuPower Taq PCR PreMix (Bioneer, Korea) was added to a final volume of 20 μl. The PCR products were electrophoresed in 1.5% agarose to confirm the results.

### Comparative Genomics

To compare the similarity of the induced prophage region of *Leu. Lactis* CBA 3262 (1391006-1428849) predicted using Prophage Hunter with other phages, BLASTn was used, and the phage genome annotation file with the highest query was downloaded from the NCBI database. Genome comparison was performed using the tblastx algorithm in the Easyfig 2.5.5 software [31] with a maximum E-value of 0.0001 and minimum identity value of 80% blast options.

## Results and Discussion

### In Silico Analyses of Prophages in *Leuconostoc* Genomes

Ten intact prophages and 24 prophage regions were identified using the PHASTER algorithm, and the genome sizes of the intact prophages ranged from 33.2 kb to 54.2 kb. Total prophage genomes accounted for 0.5 to 6% of the bacterial chromosome, which appeared to be lower compared to that of other bacterial genomes (10–20%) [17]. As examples, the phage genome of *Escherichia coli* O157:H7 strain is composed of 16%, and *Streptococcus pyogenes* contains 12% prophage genomes on the chromosome [32].

The prophage distributions on 17 kimchi-derived *Leuconostoc* strains with complete genomes reported in the PARIC database were analyzed using PHASTER. Prophage regions were identified as intact, questionable, and incomplete, according to the algorithm. Among the strains listed in Table 1, eight had 10 intact prophages (one to two prophages per strain), while 13 strains had questionable and/or incomplete prophage regions on the chromosomes. Except for *L. mesenteroides* J18, all strains had at least one prophage region, including questionable and incomplete prophage regions. Compared to kimchi-derived *Lactobacillus*, *Leuconostoc* had a relatively low number of prophage regions. *Lac. brevis* and *Lac. plantarum* strains contain up to four intact prophages [29, 33].

Cases of prophages in cryptic states that were fixed in bacterial genomes were observed among the intact prophages. Although they could be excised, these prophages could not form active particles or lyse their hosts because of mutagenesis [34]. Therefore, using the NCBI database, the essential genes coding for the full functions of phages were identified. Most of the intact prophages had essential genes, such as genes for DNA replication, packaging, morphogenesis, lysis-lysogeny, and regulation/modification modules. Among the 10 intact prophages, four phages showed frameshift mutation or defect in the essential genes and were labeled as putative cryptic phages (Table 1). First, intact prophage 1 of *Leu. citreum* WiKim 0101 consisted of pseudogenes for the MCP, terminase large subunit, and tail protein, while endolysin was not detected in intact prophage 2. Second, the tail family protein in *Leu. citreum* wikim 0096 had frameshift mutation. Lastly, in *Leu. mesenteroides* WiKim 33, replisome organizer and endolysin were incomplete. Accordingly, these strains may not be fully assembled or induced.

**Fig. 1:**
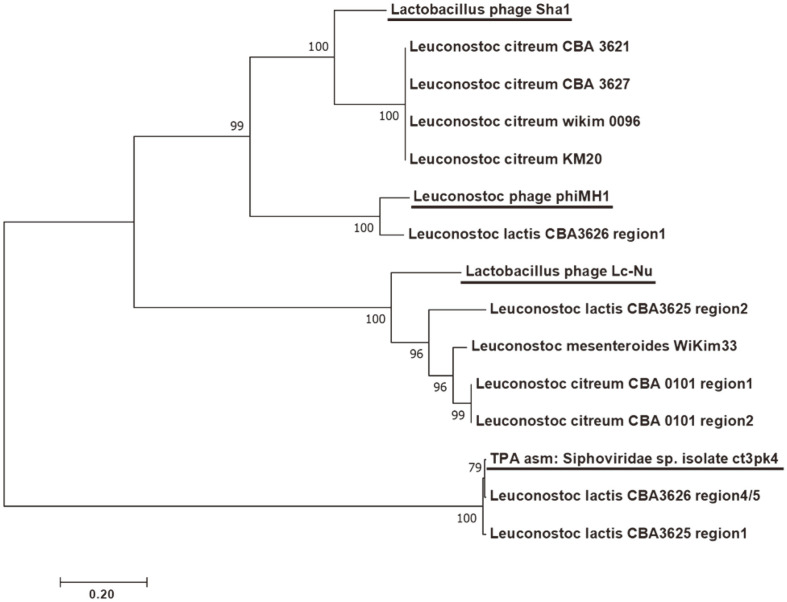
Phylogenetic tree of the *Leuconostoc* prophage region and related phages based on major capsid proteins. Underlined phages are the phages used in this study. The number on each node represents the bootstrap value (1,000 replicates).

To further characterize the prophages in kimchi-derived *Leuconostoc*, the nucleotide sequences were aligned, and a phylogenetic tree based on MCPs was generated (Fig. 1). Eleven prophages, including the induced prophage region, belonged to the *Siphoviridae* family and were similar to Lc-Nu, sha1, phiMH1, and TPA_asm phages [35-37]. Except for TPA_asm, the three phages belonged to the HK97 family [38]. However, it was difficult to analyze homologies for other morphogenesis and packaging genes because there was no similarity among the phages. Dairy *Leuconostoc* lytic phages have been classified as members of the *Siphoviridae* family; however, some phages in sauerkraut fermentations have been identified as members of family *Myoviridae* [39, 40]. The temperate phages isolated from *Leuconostoc* spp. in watery kimchi have also been reported as members of *Myoviridae* [41]. In this study, it is noteworthy that all intact prophages in *Leuconostoc* belonged to family *Siphoviridae*.

### Identification of Phage-Encoding Sie Proteins and MTase

To invade the host bacteria and integrate successfully into the genome, phages are required to overcome and adapt to host anti-phage mechanisms, such as restriction-modification (RM) systems, CRISPR-Cas immune system, abortive infection, and toxin-antitoxin systems [42]. Bacteria have been reported to have DNA MTase that transfers a methyl group from S-adenosyl-L-methionine to a target nucleotide to protect the cell from invasion by foreign DNA [43]. Phages from diverse ecosystems integrate cognate MTase-encoding genes that have the advantage of permanently overcoming the host RM hurdle. In addition, Sie proteins on host genome prophages prevent infection and multiplication of other phages by blocking DNA integration, thereby protecting the host from newly incoming phages [44].

In this study, Sie proteins and MTase from intact prophages were predicted using BlastP and TMHMM. Five prophages were predicted to have MTase and Sie proteins (Table 2). Only one intact prophage found on *Leu. lactis* CBA3625 had MTase, while the others did not harbor the gene. Meanwhile, the prophages of *Leu. citreum* CBA3621, *Leu. citreum* CBA3627, *Leu. citreum* WiKim 0096, and *Leu. lactis* CBA3626 encoded for Sie proteins. The presence of Sie proteins in the prophages might confer phage immunity to *Leuconostoc* strains over other phages, similar to *Streptococcus thermophilus* [45]. However, Sie and MTase genes on the prophages of *Lac. plantarum* showed high ratios among the strains by 80% and 50%, respectively [29]. Therefore, prophages that have these proteins may be strain-specific; thus *Leuconostoc* strains may have different characteristics in terms of evading other phages.

### Induction and Detection of the *Leu. lactis* Prophage

**Fig. 2:**
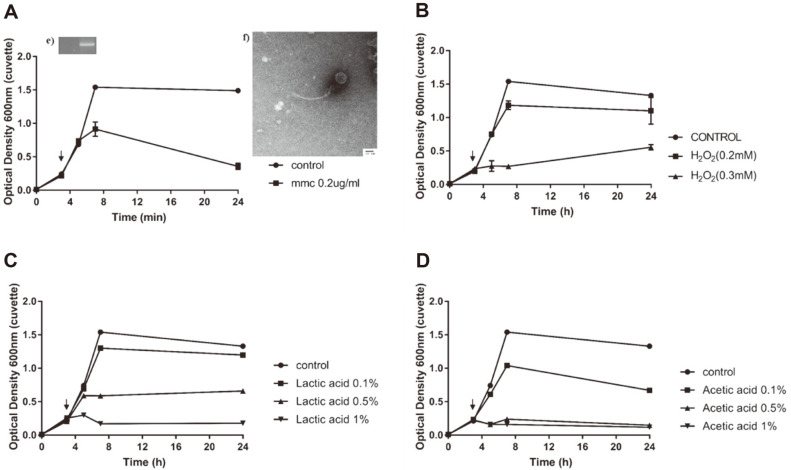
Growth curves of *Leuconostoc lactis* CBA3626 and prophage inductions using four different stresses. (**A**) Mitomycin C (mmc, 0.2 μg/ml); (**B**) H_2_O_2_ (0.2 mM, 0.3 mM); (**C**) Acetic acid (0.1 %, 0.5 %, 1 % v/v); (**D**) Lactic acid (0.1 %, 0.5 %, 1 % v/v). The control was not treated by any environmental stressor. Each stressor was added after 3 h (arrows). (**E**) In mitomycin C-concentrated supernatant, major capsid protein gene was amplified using PCR. (**F**) Induced phage particles morphology was analyzed using TEM, 50,000 ×, Scale bar; 20 nm.

**Fig. 3:**
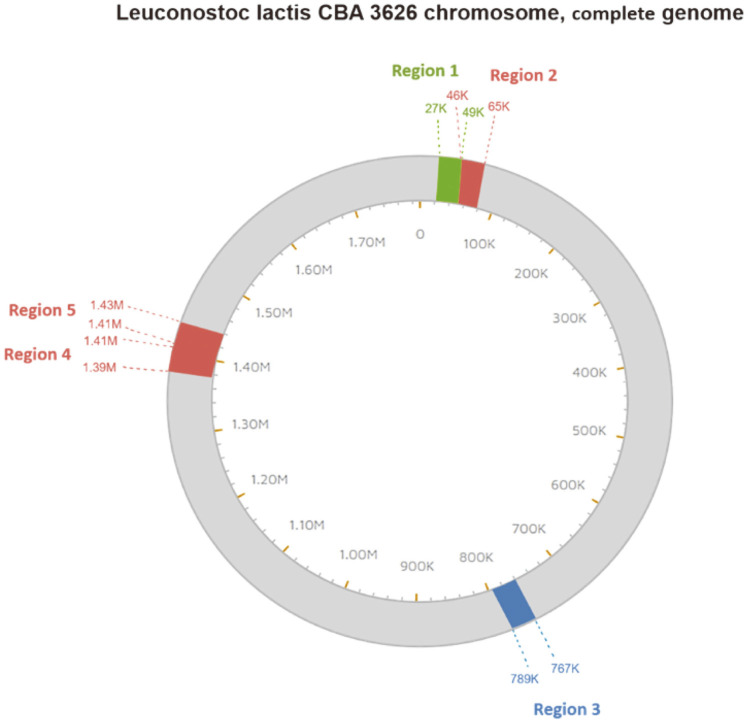
Location of prophage regions on *Leu. lactis* CBA3626 chromosome predicted using the PHASTER algorithm. Each color indicates intact, questionable, incomplete prophage regions. Green, intact prophage; blue, questionable prophage; pink, incomplete prophages.

**Fig. 4:**
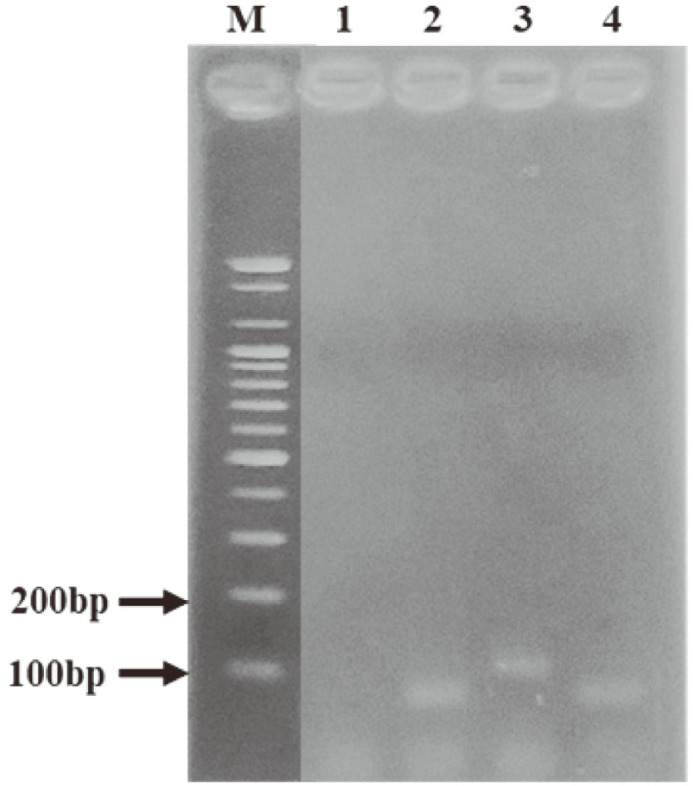
PCR analysis for the induction of two fused incomplete prophage of *Leu. lactis* CBA3626. PCR products were electrophoresed in 1.5% agarose gel. Supernatants were treated with DNase to remove the genomic DNA. G (GAPDH) gene was used as control of bacterial genomic DNA. Lane M, 100bp size marker; 1, gene amplicons for GAPDH; 2, major capsid protein; 3, endolysin; and 4, tail protein.

Among the eight strains with intact prophages, those of *Leu. citreum* CBA3621, *Leu. citreum* CBA 3627, and *Leu. lactis* CBA3626 were induced. However, prophage induction of *Leu. citreum* CBA3621 and *Leu. citreum* CBA3627 was not confirmed using PCR or TEM in all MitC concentrations. Therefore, *Leu. lactis* CBA3626 was selected for prophage induction and was induced further with various chemical stresses (Fig. 2). First, 0.2 μg/ml of MitC was added when the culture reached to 0.2 by OD_600_. After 4 h, the bacterial growth curve was different from that of the negative control, and the supernatant was harvested at 24 h. Morphology was confirmed using TEM, and phage particles were observed. The induced phage morphology exhibited an approximately 60–61 nm icosahedral head and a 132–200-nm-long, non-constrictive tail, similar to the *Siphoviridae* family (Fig. 2A). PCR amplification of the MCPs was performed to confirm which phage was induced among prophages. Unexpectedly, the MCP primers were not able to detect the intact prophages; however, two fused, incomplete prophages of Regions 4 (site 1387756–1411885) and 5 (site 1405961–1430068), approximately 42 Kbp, were detected using PHASTER analysis (Fig. 3). Another prophage prediction program of Prophage Hunter was used and suggested that the two fused, incomplete regions were one active assembly [23]. Meanwhile, the tail regions and endolysin proteins were detected using PCR (Fig. 4). Thus, induction of the prophage in *Leu. lactis* CBA3626, which might have originated from two fused regions of the incomplete prophages, was confirmed using MitC. However, the induced prophage could not confirm the plaque in any *Leuconostoc* strains, including the host.

Induction using other chemical stressors, such as acetic acid, lactic acid, and hydrogen peroxide, was performed using the same method for MitC. Although the growth patterns were similar to those in MitC induction, induction of prophages was not confirmed using spotting assay, PCR, and TEM. This result suggests that the prophage of *Leu. lactis* CBA3626 could not be induced in the kimchi environment. *Lactococcus* phages were easily detected when the dairy starter strains were induced [46], whereas *Leuconostoc* phage was observed at a relatively lower frequency [47]. Thus, these results suggest that *Leuconostoc* might not be induced well compared to *Lactococcus* or other starter strains.

### Comparative Genomics Analysis of *Leu. lactis* Prophage

**Fig. 5:**
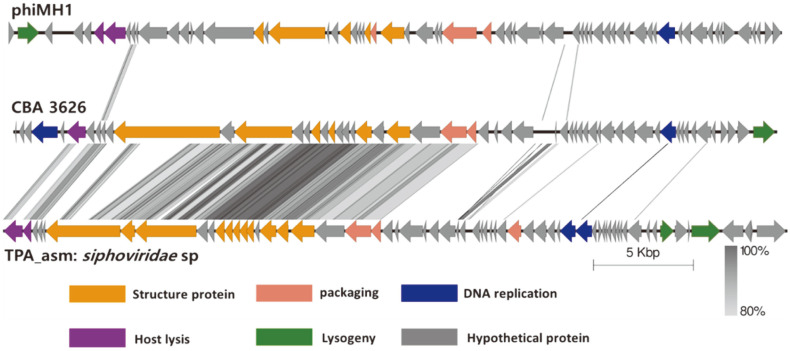
Genome comparison of induced prophage of *Leuconostoc lactis* CBA3626, phages phiMH1, and TPA_asm:*Siphoviridae* sp. isolate ct3pk4. The figure was generated using Easyfig 2.2.5. Between the genome maps, the gray regions indicate the identity values from 80 to 100%. Gene products are shown in different colors according to their functions.

Among the prophages of *Leu. lactis* CBA3626, two fused, incomplete prophages were induced using MitC and identified using PCR and TEM. Based on NCBI and BLASTp, genome comparison of the induced prophage with other *Leuconostoc* phages was performed using the representative phiMH1 [48]. Except TPA_asm, 22 reported phages on NCBI showed very low identity (< 4%) with the induced prophage. Only the TPA_asm phage derived from human metagenome research was similar to the induced phage and showed 47% homology. The structure, lysis, and packing modules were highly similar (> 84%) to the TPA_asm phage, but the genes involved in lysogen showed relatively low identities (Fig. 5). Contrarily, 11 *Leuconostoc* dairy bacteriophages were confirmed to have high similarity in morphology, replication, and packaging module [49]. Therefore, the induced prophage of *Leu. lactis* CBA3626 may be different from the reported *Leuconostoc* phages. The current data on the *Leuconostoc* phage genome are still lacking compared to *Lactobacillus* or *Lactococcus* phage genomes, so further research on *Leuconostoc* prophages should be conducted, which in turn could significantly affect the quality of the fermented kimchi.

## Supplemental Materials

Supplementary data for this paper are available on-line only at http://jmb.or.kr.

## Figures and Tables

**Table 1:** Prophages in complete *Leuconostoc* genomes predicted using the PHASTER algorithm.

Strain name (Isolation source)	Accession No.	Intact	Questionable	Incomplete	Putative cryptic phage	Phage genome (%)
*Leu. citreum*						
CBA3624 (kimchi)^*^	CP042413	-	-	3	-	1.40
CBA3623 (kimchi)^*^	CP042393	-	-	1	-	0.57
CBA3621 (kimchi)^*^	CP042410	1	-	-	-	2.23
CBA3627 (kimchi)^*^	CP042418	1	-	-	-	2.23
WiKim 0101 (kimchi)	CP046149	2	1	1	2	6.12
WiKim 0096 (kimchi)	CP066296	1	-	1	1	3.42
KM20 (kimchi)	DQ489736	1	-	1	-	2.95
*Leu. lactis*						
WiKim40 (kimchi)^*^	CP016598	-	1	3	-	2.61
CBA3625 (kimchi)	CP042387	2	-	-	-	4.34
CBA3622 (kimchi)^*^	CP042420	-	1	-	-	2.51
CBA3626 (kimchi)^*^	CP042390	1	1	3	-	6.21
*Leu. mesenteroides*						
J18 (kimchi)	CP003106	-	-	-	-	
WiKim33 (kimchi)	CP021491	1	-	-	1	2.37
CBA3628 (kimchi)	CP042404	-	1	-	-	0.91
DRC1506 (kimchi)	CP014611	-	-	1	-	0.75
DRC0211 (kimchi)	CP013016	-	-	4	-	2.19
CBA3607 (kimchi)^*^	CP046062	-	1	-	-	0.91

^*^These strains were used in this study.

**Table 2:** *Leuconostoc* prophage regions encoding methylases and Sie proteins related to phage-resistance.

Strain name	Accession No.	Methylases	Sie protein
*Leu. citreum*			
CBA3624	CP042413	-	-
CBA3623	CP042393	-	-
CBA3621	CP042410	-	1(518606..519307)
CBA3627	CP042418	-	1(1289571..1290272)
WiKim 0101	CP046149	-	-
Wikim 0096	CP066296	-	1(1044309..1044998)
KM20	DQ489736	-	-
*Leu. lactis*			
WiKim40	CP016598	-	-
CBA3625	CP042387	1(106314..107363)	-
CBA3622	CP042420	-	-
CBA3626	CP042390	-	1(1392264..1392890)
*Leu. mesenteroides*			
J18	CP003106	-	-
WiKim33	CP021491	-	-
CBA3628	CP042404	-	-
DRC1506	CP014611	-	-
DRC0211	CP013016	-	-
CBA3607	CP046062	-	-
