# Supplementary material for: Characterization of Prophages in Leuconostoc Derived from Kimchi and Genomic Analysis of the Induced Prophage in Leuconostoc lactis
Source: J Microbiol Biotechnol. 2021 Dec 23;32(3):333–40. doi: 10.4014/jmb.2110.10046 (PMC9628853; doi:10.4014/jmb.2110.10046)
Supplement: Supplementary file 1 [file jmb-32-3-333-supple.pdf]

J. Microbiol. Biotechnol.
https://doi.org/10.4014/jmb.2110.10046

Supplementary Table 1. PCR primer sequences based on major capsid proteins of intact, incomplete, and questionable *Leu. lactis* CBA3626 prophages.

Primer	Sequence	Size (bp)
MCP1_F	GATAACGCTGACGCTTTGATGCA	725
MCP1_R	ACGTTGGTTGATGACTTGAAGACAAT	
MCP2_F	GCGACAAAGATAACAGAGGTACG	550
MCP2_R	CTGTTAATCCAGATATTGCTAAGGCA	
MCP3_F	TAGCAAACTTGATCGACCCAGAAG	287
MCP3_R	CAGTCTTGTCTGGTTTGGGTGAT	

Supplementary Table 2. PCR primers based on major capsid protein (MCP) and endolysin (LYS) tail protein (Tail), and glyceraldehyde 3-phosphate dehydrogenase (GAPDH) were used to identify induced prophage of *Leu. Lactis.*

Primer	Sequence	Size (bp)
GAPDH_F	TTGGTGACACACACGGTACA	100
GAPDH_R	TCGTTATCGTACCATGCAGC	
MCP_F	GCGTGACATCTTTGCTGAAG	96
MCP_R	GCATCGTTGTAGATAGCCAC	
LYS_F	ACGAGGTAACCAAGCTGCAA	126
LYS_R	CCGTAGTTGCGGTACTTGAT	
Tail_F	GTGATGACCGTAAGGTTTGG	98
Tail_R	ACCATCACCGGCAACGATTT	
